# Identification and Characterization of microRNAs and Their Predicted Functions in Biomineralization in the Pearl Oyster (*Pinctada fucata*)

**DOI:** 10.3390/biology8020047

**Published:** 2019-06-17

**Authors:** Songqian Huang, Yuki Ichikawa, Kazutoshi Yoshitake, Shigeharu Kinoshita, Yoji Igarashi, Fumito Omori, Kaoru Maeyama, Kiyohito Nagai, Shugo Watabe, Shuichi Asakawa

**Affiliations:** 1 Graduate School of Agricultural and Life Sciences, The University of Tokyo, Bunkyo-ku, Tokyo 113-8657, Japan; huangsongqian0115@gmail.com (S.H.); dedeneko@gmail.com (Y.I.); akyoshita@g.ecc.u-tokyo.ac.jp (K.Y.); akino@mail.ecc.u-tokyo.ac.jp (S.K.); aiga@mail.ecc.u-tokyo.ac.jp (Y.I.); 2 Mikimoto Pharmaceutical CO., LTD., Kurose 1425, Ise, Mie 516-8581, Japan; oomori.353@mikimoto-cosme.com (F.O.); maeyama.511@mikimoto-cosme.com (K.M.); 3 Pearl Research Laboratory, K. MIKIMOTO & CO., LTD., Osaki Hazako 923, Hamajima, Shima, Mie 517-0403, Japan; k-nagai@mikimoto.com; 4 School of Marine Biosciences, Kitasato University, Minami-ku, Sagamihara, Kanagawa 252-0313, Japan; swatabe@kitasato-u.ac.jp

**Keywords:** miRNA, target prediction, biomineralization-related genes, biomineralization, *Pinctada fucata*

## Abstract

The biological process of pearl formation is an ongoing research topic, and a number of genes associated with this process have been identified. However, the involvement of microRNAs (miRNAs) in biomineralization in the pearl oyster, *Pinctada fucata*, is not well understood. In order to investigate the divergence and function of miRNAs in *P. fucata*, we performed a transcriptome analysis of small RNA libraries prepared from adductor muscle, gill, ovary, and mantle tissues. We identified 186 known and 42 novel miRNAs in these tissues. Clustering analysis showed that the expression patterns of miRNAs were similar among the somatic tissues, but they differed significantly between the somatic and ovary tissues. To validate the existence of the identified miRNAs, nine known and three novel miRNAs were verified by stem-loop qRT-PCR using *U6* snRNA as an internal reference. The expression abundance and target prediction between miRNAs and biomineralization-related genes indicated that miR-1990c-3p, miR-876, miR-9a-3p, and novel-3 may be key factors in the regulatory network that act by controlling the formation of matrix proteins or the differentiation of mineralogenic cells during shell formation in mantle tissue. Our findings serve to further clarify the processes underlying biomineralization in *P. fucata*.

## 1. Introduction

The pearl oyster (*Pinctada fucata*) is a well-studied organism, owing to the economic potential of pearl production as well as the fascinating biology of mollusks. *P. fucata* is also a representative experimental model for biomineralization analysis [1,2]. A number of genes involved in biomineralization have been identified, and their functions in pearl and shell formation have been clarified [3,4,5,6,7]. Recently, transcriptomics [8,9], proteomics [10,11,12], genomics [1,2,13,14], and gene interference techniques [15,16] have been used to investigate genetic components of shell and pearl formation in mollusks. 

MicroRNAs (miRNAs) are a class of small endogenous noncoding RNAs, 20–25 nucleotides (nt) in length. They are embedded within the stem regions of hairpin transcripts that exist in a wide range of invertebrates and vertebrates [17]. A single miRNA can regulate hundreds of target genes. Up to 30% of human protein coding genes may be regulated by miRNAs [18]. miRNAs play a vital role in the regulation of gene expression at the post-transcriptional level, especially for signaling pathways involved in cellular development, proliferation, apoptosis, oncogenesis, and differentiation [19,20]. miRNAs negatively regulate gene expression through sequence-specific interactions with the 3′ untranslated regions (UTRs) of target genes, and thereby cause translational repression or mRNA destabilization [21,22]. Prior research has shown that some miRNAs, such as miR-223, miR-125b and, miR-302a, participate in the control of biomineralization during bone formation in animals [23,24,25].

Mollusks are the second largest phylum in Metazoa, yet very few studies have addressed the diversity and function of miRNAs in mollusks. So far, five miRNAs have been identified from *Holiotis rufescens*, 60 miRNAs from *Lottia gigantean* [26], 258 miRNAs from *Pinctada martensii* [27], 199 miRNAs from *Crassostrea gigas* [28], and 289 miRNAs from *Crassostrea hongkongensis* [29]. Moreover, a computational prediction of candidate miRNAs was performed for the *Patella vulgata* [30] and *P. martensii* [31] genomes. In addition, prior work revealed that miR-2305 participates in nacre formation by targeting *pearlin* in *P. martensii* [32]. However, data regarding the involvement of miRNAs in pearl formation remain limited.

To obtain various miRNAs and explore their potential functions in biomineralization, we constructed and sequenced eight small RNA libraries prepared from adductor muscle, gill, mantle, and ovary tissues of two *P. fucata*. The genomic and transcript sequences of *P. fucata* are very helpful for performing functional analysis of miRNAs in this organism. The clarification of the functions of miRNAs in *P. fucata* will provide new insights to further understand the molecular mechanisms of biomineralization. In addition, this will provide useful information for further research into various biological processes in *P. fucata*. The aims of this study were (1) to identify known and novel miRNAs of *P. fucata*, (2) to clarify the expression profile of identified miRNAs in the tissues examined, (3) to predict genes targeted by the miRNAs, and (4) to identify the miRNAs involved in biomineralization.

## 2. Materials and Methods

### 2.1. Ethics

This study was conducted in strict accordance with the recommendations in the Guide for the Care and Use of Laboratory Animals of the University of Tokyo. All efforts were made to minimize the suffering of the animals.

### 2.2. Small RNA Isolation and cDNA Library Construction

Two female pearl oysters (approximately two years old) were collected from Mikimoto Pearl Research Institution Base, Mie Prefecture, Japan in 2014. Adductor muscle (Ad for short), gill (Gi for short), mantle (Ma for short), and ovary (Ov for short) tissues were collected from the pearl oysters. Eight RNA samples in total were extracted from the tissues, and small RNA extraction was performed following the protocol of the mirVana miRNA Isolation Kit (Life Technologies, California, USA). In brief, the sequencing libraries were constructed as per the Ion Total RNA-seq Kit v2 small RNA libraries construction protocol (Life Technologies, California, USA). This was followed by adapter ligation, synthesis of cDNA by reverse transcription, purification of the small RNA fraction, and amplification by PCR to construct cDNA libraries. An Agilent 2200 TapeStation (Agilent Technologies, Waldbronn, Germany) was used to determine the cDNA concentration. The cDNA libraries were then used to prepare a template for loading onto the analysis chip, according to the Ion PI™ Template OT2 200 Kit v3 protocol (Life Technologies, California, USA). Sequencing was then performed following the manufacturer’s protocol for the Ion PI^TM^ Sequencing 200 Kit v3 (Ion Proton Sequencer and Ion Proton Server, Life Technologies).

### 2.3. Small RNA Sequence Analysis

The raw sequencing data were saved as FASTQ files. We first filtered out the reads with low quality, containing poly-A tails, and those outside the range of 15–31 nt length (Step 1). After filtering, quality and length distribution statistics for these sequencing data were examined. Then, the filtered reads were evaluated by a homology search using basic local alignment search tool (BLAST) against the NCBI non-coding RNA database [33] and Rfam database [34] to remove rRNA, tRNA, snRNA, and snoRNA sequences (Step 2). Subsequently, the filtered reads were mapped onto the *P. fucata* genome by bowtie (-v1) (Step 3) [35]. Then, miRNA identification was performed by comparing the sequences with the known mature miRNAs and miRNA precursors in miRBase 22.0 [36]. Unannotated sequences that failed to match with the sequences in the databases above were analyzed by miRDeep2 to predict novel miRNA candidates [37]. The base bias composition at each nucleotide position of all identified miRNAs was analyzed. The genome of *P. fucata* (version 2.0) [2] was used as the reference genome for miRNA precursor prediction.

### 2.4. Analysis of Differentially Expressed miRNAs among Tissues

The total miRNA reads of each library were used for normalization to analyze differentially expressed miRNAs between two tissues’ libraries. The miRNA counts were normalized according to the reads per million reads (RPM) method. The expression levels of known and novel miRNAs in the various tissues were compared to identify differentially expressed miRNAs. DESeq2 software (version 1.14.1) [38] was used to analyze the differences in the expression levels of the miRNAs between two tissues with the criteria: |log2(FoldChange)| > 1 and *p*-adj < 0.05. Six paired comparisons were evaluated by DESeq2, including Ad vs. Gi, Ad vs. Ov, Ad vs. Ma, Gi vs. Ov, Gi vs. Ma, and Ov vs. Ma. Each tissue had two individuals as replicates.

### 2.5. Target Analysis for miRNAs and Biomineralization-Related Genes

Complete sequences of 105 biomineralization-related genes from the *Pinctada* genus were downloaded from NCBI GenBank [39], and the 3′ UTRs were used for miRNA-target prediction. Target predictions for miRNAs and biomineralization-related genes were analyzed by RNAhybrid [40], miRanda [41] and RNA22 [42]. For the RNAhybrid algorithm, the free energy (e) and *p*-value (*p*) were used to estimate the interaction between miRNAs and mRNAs. The conditions for functional targets were e < −20 kcal/mol and *p* < 0.05. Functional target genes of miRNAs identified using the miRanda algorithm were restricted by the conditions of score > 100 and free energy < −10 kcal/mol. The RNA22 algorithm was used to determine the most favorable hybridization site between miRNA and mRNA with a sensitivity of 63%, specificity of 61%, and seed size of 7, allowing a maximum of one unpaired base in the seed.

### 2.6. Validation of P. fucata miRNAs by Stem-Loop qRT-PCR

Stem-loop qRT-PCR analysis [43] was employed to validate and determine the specific expression of miRNAs in *P. fucata*. Ten adult female *P. fucata* were obtained for tissue collection from Mikimoto Pearl Research Institution Base in May 2018. Total RNA was isolated from adductor muscle, gill, ovary, and mantle tissues using an RNeasy Mini Kit (QIAGEN, Maryland, USA). RNA quality and purity were assessed using RNA ScreenTape (Agilent, California, USA). Each RNA sample (0.5 μg) was reverse transcribed into cDNA using a cDNA Synthesis Kit (TaKaRa, Shiga, Japan) as per the manufacturer’s instructions. The stem-loop primer was used for small RNA reverse transcription; qRT_PCR forward primer and common reverse primer were used to amplify small RNA sequences. PCR assays were carried out in a quantitative thermal cycler (Applied Biosystems 7300 Real-Time PCR System) (Life Technologies) in a 20 μL reaction volume containing 10 μL SYBR Premix Ex Taq II (TaKaRa), 2 μL cDNA, 0.4 μL ROX Reference Dye (50×) (TaKaRa), 6 μL sterile distilled water, and 0.4 μM of each primer. Primer sequences are listed in Table 1. The thermal program included 95 °C for 30 s, 40 cycles of 95 °C for 5 s, 60 °C for 31 s, and a dissociation stage of 95 °C for 15 s, 60 °C for 60 s and 95 °C for 15 s. The expression of each miRNA quantified by stem-loop qRT-PCR was presented as n = 10 done for three replicates, and *U6* snRNA was used as the internal reference for the stem-loop qRT-PCR. Ct values were represented by the mean values of three independent replicates, and the relative expression levels were calculated using the 2^−∆∆Ct^ method [44].

### 2.7. Data Accessibility

The raw sequencing reads obtained in the present study are deposited at DNA Data Bank of Japan (DDBJ) Sequence Read Archive (DRA) under submission accession number DRA006953.

## 3. Results

### 3.1. Overview of Small RNA Library Sequencing

**Figure 1:**
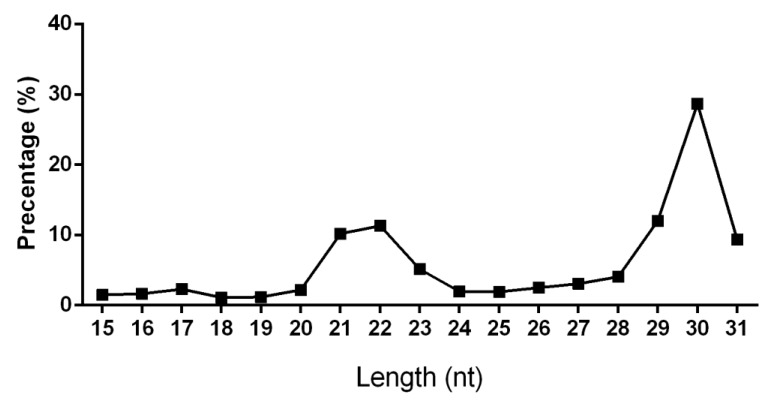
Length distribution of total filtered reads in *P. fucata*.

Eight small RNA libraries from the adductor muscle (Ad), gill (Gi), ovary (Ov), and mantle (Ma) tissues of two individual *P. fucata* were sequenced by Ion Proton sequencing to survey the miRNA diversity in *P. fucata*. In total, 50.32 million raw reads were obtained from all libraries (Table 2). After the removal of low-quality sequences, adaptor sequences, poly-A tail sequences, and sequences shorter than 15 nt or longer than 31 nt, 41.72 million reads remained for the statistical analysis of sequence length (Figure 1 and Table 2, Step 1). The length distribution of each tissue library is shown in Figure S1. Two obvious small RNA peaks with different lengths were found in the sequencing data. After removing known RNAs and genome unmapped reads, 33.26 million reads (66.1% of raw reads) were remaining for miRNA identification in this study.

### 3.2. Identification of Known and Novel miRNAs

To identify known miRNAs in *P. fucata*, we compared our dataset with the known miRNAs in miRBase 22.0. First, the remaining sequencing reads were mixed for miRNA identification. A read found more than five times was considered as a possible miRNA in *P. fucata*. Allowing no more than one mismatch between sequences, 186 known miRNAs were identified (Table S1). We then analyzed the abundance of the known miRNA, which revealed a large distribution in the expression levels. The read numbers of known miRNAs ranged from 1 to 1359669 in a single library, indicating that not only highly expressed miRNAs but also weakly expressed miRNAs were present in *P. fucata*.

One of the important characteristics that distinguish miRNAs from other endogenous small RNAs is the capacity of the precursor miRNA sequences to fold back into a canonical stem-loop hairpin structure [27]. The genomic data of *P. fucata* were used for miRNA precursor identification and were further parsed through the miRDeep2 software for the prediction of precursor sequences and secondary structures. A total of 280 sequences could form proper secondary hairpins and were considered miRNA precursors. Moreover, 186 known and 42 novel miRNAs were identified based on expression levels and precursor identification. The base bias at each position of all identified miRNAs is shown in Figure S2. The majority of miRNAs tended to start with 5′-U, which is consistent with typical miRNA sequence patterns [17]. Of these miRNAs, 38 (32 known and six novel miRNAs) have multiple (from two to five) precursor sequences (Table S1). For example, miR-9649 and novel-18 have five and three precursors, respectively.

### 3.3. Expression Levels of miRNAs in P. fucata

**Figure 2:**
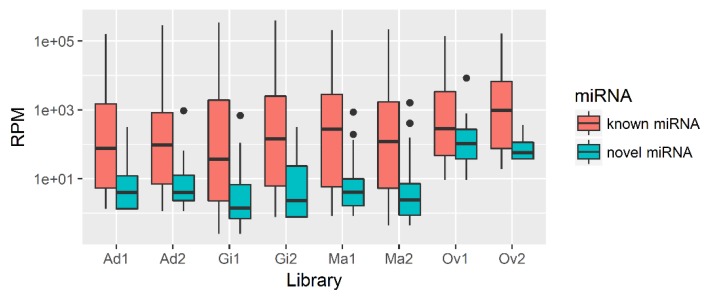
Expression density of known and novel microRNAs (miRNAs) in *P. fucata*. Ad: adductor muscle; Gi: gill; Ma: mantle; Ov: ovary.

Among 228 miRNAs identified, 165, 198, 159 and 185 miRNAs were found in adductor muscle, gill, ovary and mantle tissues, respectively. Of these, 119 miRNAs (52.19% of total miRNA) were ubiquitously expressed in all examined tissues. On the contrary, 19 miRNAs (11.95% of ovary miRNAs) and eight miRNAs (4.04% of gill miRNAs) were uniquely expressed in ovary and gill tissues, respectively (Figure S3). One miRNA, miR-8871, was also uniquely expressed in mantle tissues, albeit with a low expression level. The expression levels of *P. fucata* miRNAs were calculated by reads per million reads (RPM), and these data are shown in Table S2. The novel miRNAs were expressed at lower levels than the previously known miRNAs (Figure 2).

**Figure 3:**
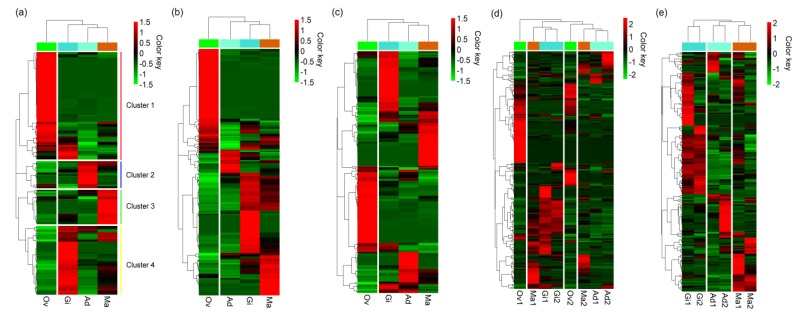
Heat map of expression levels of miRNAs in *P. fucata* somatic and ovary tissues, based on (**a**) average expression levels of the two individuals, (**b**) individual expression levels of individual 1, (**c**) individual expression levels of individual 2, (**d**) all examined samples of the two individuals, and (**e**) six somatic samples of the two individuals. The details regarding miRNA expression are shown in Table S2. RPM: reads per million reads. Ad: adductor muscle; Gi: gill; Ov: ovary; Ma: mantle.

Among the miRNAs identified in *P. fucata*, miR-279 showed the highest expression levels in gill and mantle tissues, whereas miR-100 and miRNA-549a showed the highest expression levels in adductor muscle and ovary tissues. Let-7a, miR-184-3p, miR-71, and miR-87a also demonstrated high expression in all examined tissues in *P. fucata*, while miR-133a, miR-549a, and miR-1990c-3p were highly expressed in adductor muscle, ovary and mantle tissues, respectively. The expression patterns of miRNAs in ovary tissue showed rather different features from those in the somatic tissues (Figure 3). In Figure 3a, four clusters of miRNAs are shown based on their expression patterns, in which each cluster corresponds to highly and specifically expressing miRNA groups in each tissue based on the average expression levels of two replicates. The expression pattern of ovary tissue differed from somatic tissues, and 24 (57.14% of novel miRNAs) novel miRNAs were highly expressed in ovary tissue. Essentially identical clustering patterns were observed based on individual expression levels (Figure 3b,c). However, when we examined clustering patterns of the eight samples (four tissues of the two individuals), mixed and complicated patterns were observed in somatic and ovary tissues (Figure 3d). Then, we examined clustering patterns of the six somatic samples, in which a pair for each tissue clustered together, indicating that the expression of miRNAs in the somatic tissues showed similar patterns between the two individuals (Figure 3e). The principal component analysis (PCA) also showed large differences in miRNA expressions between ovary and somatic tissues (Figure S4). Data regarding the miRNA expressions as obtained from high-throughput sequencing are shown in Table S2. For mantle tissues, eight miRNAs, including miR-1493, miR-1993, miR-9a-3p, miR-9b-3p, novel-3, miR-876, miR-1990c-3p, and miR-10a, were significantly highly expressed when compared with the other three tissues (Figure S5).

### 3.4. Validation of miRNA by Stem-Loop qRT-PCR

**Figure 4:**
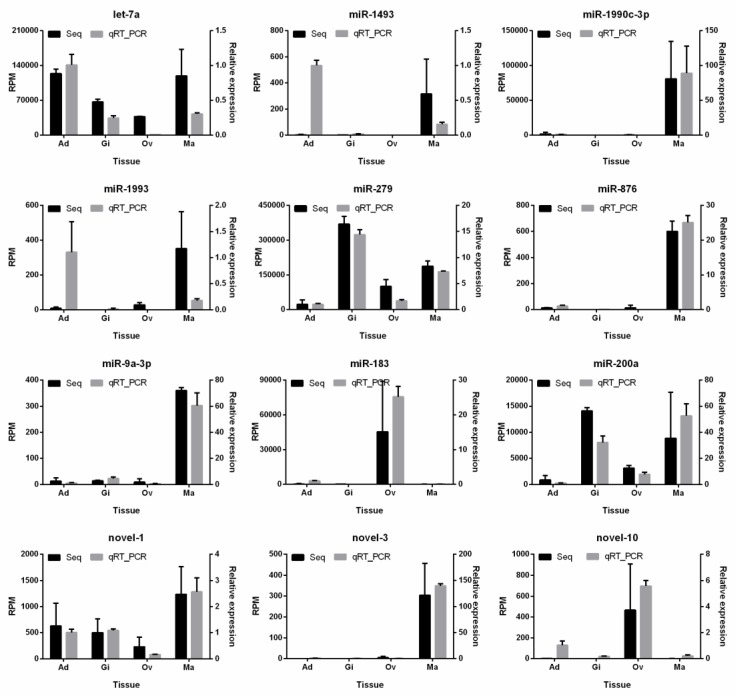
qRT-PCR validation of miRNAs expressed in *P. fucata* somatic and ovary tissues. Ad: adductor muscle; Gi: gill; Ov: ovary; Ma: mantle; RPM: reads per million reads; Seq: high-throughput sequencing.

The total RNA extracted from adult *P. fucata* was subjected to stem-loop qRT-PCR to validate the authenticity of miRNA expression shown by the transcriptome analysis. Twelve miRNAs, including nine known miRNAs and three novel miRNAs, were selected for the checking of miRNA expression. Of those, six miRNAs, namely miR-1493, miR-1990c-3p, miR-1993, miR-876, miR-9a-3p, and novel-3, were selected because of their high expression in mantle tissues. The other miRNAs were selected randomly. All of these miRNAs could be readily detected by stem-loop qRT-PCR. Except for inconsistency in the expression of miR-1493 and miR-1993 in adductor muscle between the transcriptome and qRT-PCR data, the other ten miRNAs demonstrated consistent levels of relative expression by qRT-PCR when compared with those obtained from the high-throughput sequencing data (Figure 4). Let-7a, novel-1, miR-279, and miR-200a were ubiquitously expressed in all examined tissues, while miR-279 and miR-200a were highly expressed in gill, ovary, and mantle tissues. Among these miRNAs, miR-1990c-3p, miR-876, miR-9a-3p, and novel-3 were highly expressed in mantle tissues, while miR-183 and novel-10 were highly expressed in ovary tissue when compared with the other three tissues.

### 3.5. Functional Prediction of miRNA in Biomineralization

**Figure 5:**
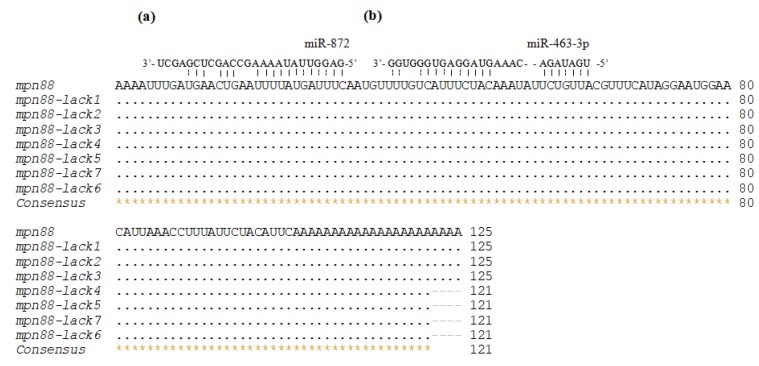
The predicted interactions of miR-872 and miR-463-3p with the 3′ untranslated regions (UTRs) of *MPN88* family genes. The target sites of miR-872 (**a**) and miR-463-3p (**b**) in the 3′UTRs of eight *MPN88* family genes identified by RNA22 and RNAhybrid, respectively.

To elucidate the functions of the identified miRNAs in biomineralization, the putative target associations between miRNAs and the biomineralization-related genes were analyzed using three informatics software packages, RNAhybrid, miRanda, and RNA22, which were programmed to predict the miRNA:mRNA interaction sites. One hundred and five biomineralization-related genes from the *Pinctada* genus with completed 3′UTR sequences were downloaded from the NCBI database. As shown in Table S3, 248, 323, and 577 miRNA:mRNA interaction sites were predicted by RNAhybrid, miRanda, and RNA22, respectively. The results also demonstrated that the majority of these genes could be targeted by more than one miRNA. For example, *Fam20c* could be targeted by 27 miRNAs, of which twenty-five were known miRNAs and two were novel miRNAs. The predicted interaction sites for the same miRNA:mRNA pair varies widely among the software packages used. For example, when RNA22 was employed, the MPN88 family genes were predicted to be regulated by miR-872 (Figure 5a), while RNAhybrid predicted that MPN88 family genes were also regulated by miR-463-3p (Figure 5b).

Of these predictions, twenty-two genes are shown to be regulated by multiple miRNAs by all three programs (Table S3). On the other hand, no interaction sites were predicted in eight genes by all three programs. Moreover, some miRNAs are predicted to regulate multiple genes, whereas some genes are predicted to be regulated by multiple miRNAs. For example, while let-7a was predicted to regulate 13 genes, including *BMP3/7, CaM, Cathepsin-B, GRP, Nacrein, PfCHS1, PFMG6, PFMG7, Pif177-like*, and *shematrin-5/6/8*, the gene *BMP3*, a subgroup of the bone morphogenetic protein (BMP) family, could be regulated by either let-7a, miR-133a, or miR-9007. Among these potential target genes, most of them are shell matrix proteins, such as *GRP*, *KRMP, Linkine*, *Pif177-like*, *PFMG, Prismalin-14*, *SGMP1*, *Shematrins* etc., which constitute the basic calcium carbonate and organic matrices formed during the biomineralization process. Moreover, many protein kinases (*ALP, CaM*, and *MMP*) and transcription factors (*OCT4*) are also regulated by miRNA according to target site predictions (Table S3).

**Figure 6:**
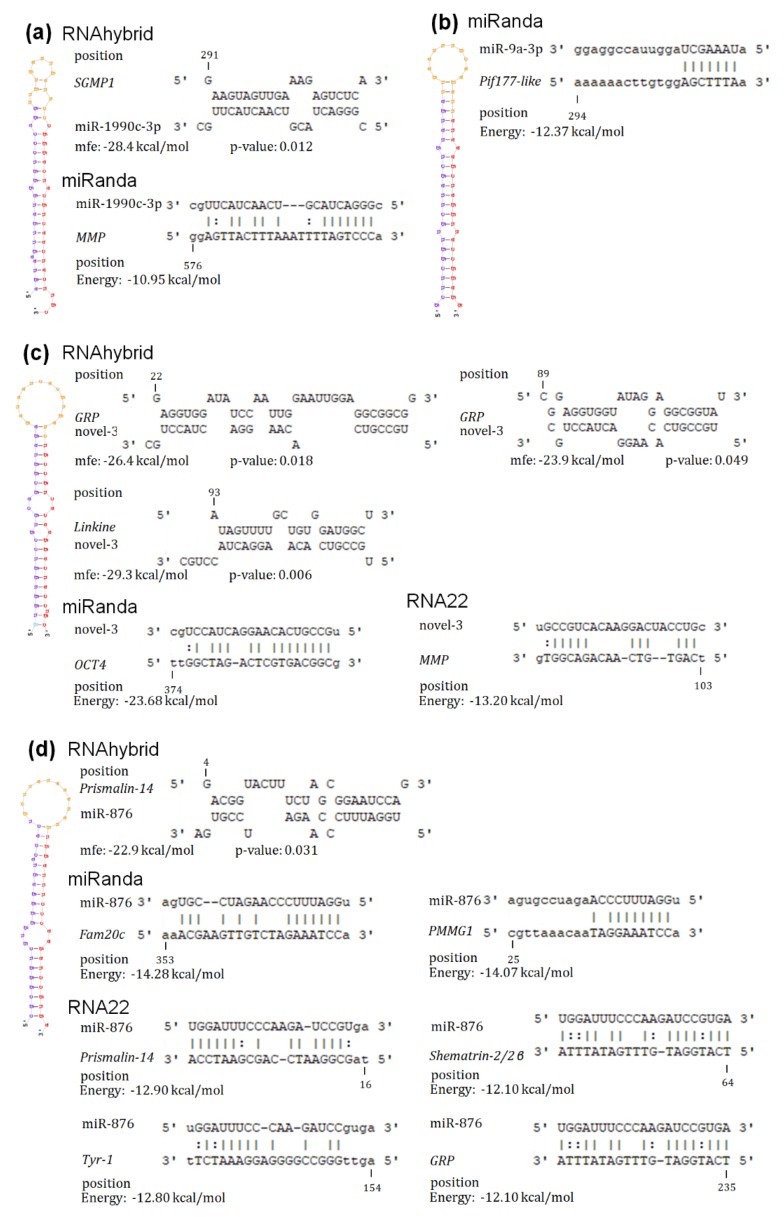
Hairpin structure of miRNAs and the potential target sites. (**a**) The potential target sites between miRNA-1990c-3p and *MMP* by miRanda, and between miRNA-1990c-3p and *SGMP1* by RNAhybrid. (**b**) The potential target site between miR-9a-3p and *Pif177-like protein* by miRanda. (**c**) The potential target sites between novel-3 and *GRP*, between novel-3 and *Linkine* by RNAhybrid, and between novel-3 and *OCT4* by miRanda, and between novel-3 and *MMP* by RNA22. (**d**) The potential target sites between miR-876 and *Prismalin-14* by RNAhybrid and RNA22, and between miR-876 and *Fam20c*, between miR-876 and *PMMG1* by miRanda, between miR-876 and *GRP*, between miR-876 and *shematrin-2/2β*, and between miR-876 and *Tyr-1* by RNA22. The position of target genes is numbered from the 3′UTR starting position.

miR-1990c-3p, miR-876, miR-9a-3p, and novel-3 were highly expressed in mantle tissues, and each of these miRNAs has multiple target genes related to biomineralization. Therefore, these miRNAs are very likely to be involved in the biomineralization process. Indeed, miR-1990c-3p was predicted to regulate *MMP* and *SGMP1*; miR-9a-3p regulated *Pif177-like*; miR-876 regulated *Fam20c, GRP, PMMG1, Prismalin-14, shematrin-2/2β*, and *Tyr-1*; and novel-3 regulated *GRP, Linkine, MMP*, and *OCT4* (Figure 6).

## 4. Discussion

Prior research has demonstrated that miRNAs are a major class of post-transcriptional regulatory molecules that have critical functions in diverse biological processes [45]. Abundant miRNAs have been identified in various organisms, while only a small number of miRNAs have been identified and undergone functional prediction in mollusks such as *P. fucata* [31]. In the present study, two main peaks of different sizes of small RNAs were observed in the distribution of small RNA lengths by high-throughput sequencing, with small RNAs with a length of 21–23 nt likely representing miRNAs, while those 29–31 nt in length mainly represented putative Piwi-interacting RNAs (piRNA) [46]. Separate analyses were conducted on these two different sets of small RNAs (we focused on the miRNA fraction in this study).

A total of 228 miRNAs, including 186 known and 42 novel miRNAs, were identified by high-throughput sequencing in *P. fucata*. To gain a better understanding of miRNA expression in *P. fucata*, the normalized RPM of each miRNA was used to compare miRNA expression among tissues. More than 50% of miRNAs were commonly expressed in all tissues, while a few miRNAs were specifically expressed in a single tissue. Clustering analysis and PCA of miRNA expression patterns, based on average expression levels, clustered the somatic tissues together, indicating that the miRNA expression patterns in *P. fucata* differ between the somatic and ovary tissues. These results demonstrate that the expression of miRNAs in *P. fucata* is both common and tissue-specific, which may play an important role in development, differentiation, and biological processes. The results of the eight samples in Figure 3d showed mixed and complicated clustering patterns. This result might be caused by the perturbation of relatively differential expression levels of miRNAs in ovaries and mantles between the two individuals.

Among these 228 miRNAs, many known miRNAs were highly expressed in *P. fucata*. miR-279 has been observed as being well preserved in arthropods, but is rarely observed in vertebrates [47]. Previous studies showed that the miR-279 family is involved in the proper development of CO_2_ sensory neurons and the maintenance of circadian rhythm in *Drosophila* [48,49]. miR-100 was also ubiquitously expressed in all examined tissues by high-throughput sequencing (Table S2). In previous studies, it was considered a tumor suppressor miRNA in patient tissue, and the decreased expression of miR-100 appears to play an important role in the development and progression of disease and may contribute to reduced sensitivity to ionizing radiation [50,51]. Since no predicted interaction site was identified between these two miRNAs and the biomineralization-related genes (Table S3), miR-279 and miR-100 may be involved outside of the biomineralization process in *P. fucata*. Let-7a was also found in most animals, suggesting that these miRNAs, which regulate cell proliferation and differentiation, are evolutionarily well conserved [52,53].

In addition, 42 novel miRNAs were identified in *P. fucata*. The expression density of novel miRNAs was lower than that of the majority of the known miRNAs. This result is consistent with previous observations suggesting that novel miRNAs are often expressed at lower levels than known miRNAs [27]. Many of the novel miRNAs (57.14%) are likely specialized for reproductive functions given their tissue-specific biases in expression. This finding indicates that these novel miRNAs may have not general but specific functions. Further experiments could provide further insight into the functions of these novel miRNAs in *P. fucata*.

To verify the authenticity of miRNAs identified by Ion Proton sequencing, we performed stem-loop qRT-PCR to detect and measure the relative expression levels of miRNAs in *P. fucata*. All of the selected miRNAs were easily detected by stem-loop qRT-PCR. Most of the miRNA expression levels obtained by stem-loop qRT-PCR are consistent with those measured by Ion Proton sequencing, except for miR-1493 and miR-1993 in adductor muscle. This inconsistency may be caused by stem-loop qRT_PCR primer amplification efficiency, sequencing errors during the complex sequencing protocols [27] or differences among individual *P. fucata*. Nevertheless, the overall results of sequencing technology and stem-loop qRT-PCR in our study are highly consistent, suggesting that expression analysis was largely able to reveal accurate miRNA expression levels in *P. fucata*. 

The biomineralization process is very complex and precise, and the expression of each related protein is subject to fine regulation [31]. Therefore, it would be beneficial to gain a better understanding of the regulators involved in biomineralization. Generally, miRNAs are fine regulators that function by silencing the expression of endogenous genes. In the present study, hundreds of interaction sites between miRNAs and biomineralization-related genes were predicted by RNAhybrid, miRanda, and RNA22, which were widely used in many reports, especially in the initial research when there was little knowledge about the miRNAs in this species [54,55,56]. Many biomineralization-related genes, such as *ALP, BMP, CaM, GRP, KRMP, MMP, N16, OCT4, Pif177*, *PFMG, Prismin, SGMP*, *shematrin*, etc., are regulated by miRNA according to the target sites’ prediction. Shematrins are synthesized in the mantle edge and secreted into the prismatic layer of the shell, where the protein family is thought to provide a framework for calcification in *P. fucata* [5]. Matrix metalloproteinases (*MMP*s), which can be regulated by miR-1990c-3p, digest proteins to prepare the peptides that contribute to calcium carbonate crystallization [57]. The transcription factor Pf-POU3F4 (*OCT4*), which is predicted to be regulated by novel-3, regulates the expression of the matrix protein genes *Aspein* and *Prismalin-14* [58]. These results suggest that miRNAs are involved in the biomineralization process, not only through targeting the shell matrix protein, but also through the regulation of protein kinases and transcription factors. Here, we provided an important set of resources for future studies of the miRNA-dependent regulation of expression in the biomineralization process that forms pearls.

## 5. Conclusions

A total of 228 miRNAs were identified in *P. fucata* by high-throughput sequencing in the present study. Novel miRNAs demonstrate weaker expression than previously known miRNAs. Clustering analysis showed that the expression patterns of miRNAs were similar within the somatic tissues, but differed significantly between the somatic and ovary tissues. Hundreds of potential target sites of miRNAs and target genes were detected using multiple software tools. miR-1990c-3p, miR-876, miR-9a-3p, and novel-3, which were highly expressed in mantle tissues, may play a core role in biomineralization by regulating the formation of matrix proteins or protein kinase and transcription factor genes. These results further clarify the range of miRNAs found in *P. fucata* and their possible functions in biomineralization in mollusks.

## Figures and Tables

**Table 1:** Primers used in the present study.

Small RNA	Stem-Loop Primer	qRT-PCR Forward Primer
let-7a	CTCAACTGGTGTCGTGGAGTCGGCAATTCAGTTGAGAACTATAC	ACACTCCAGCTGGGTGAGGTAGTAGGTTGT
miR-1493	CTCAACTGGTGTCGTGGAGTCGGCAATTCAGTTGAGACTGATGT	ACACTCCAGCTGGGAGAACTGTGTATGGAC
miR-1990c-3p	CTCAACTGGTGTCGTGGAGTCGGCAATTCAGTTGAGGCAAGTAG	ACACTCCAGCTGGGCGGGACTACGTCAACT
miR-1993	CTCAACTGGTGTCGTGGAGTCGGCAATTCAGTTGAGTCTCGTGA	ACACTCCAGCTGGGTATTATGCTGTTATTC
miR-279	CTCAACTGGTGTCGTGGAGTCGGCAATTCAGTTGAGGGATGAGT	ACACTCCAGCTGGGTGACTAGATCCACAC
miR-876	CTCAACTGGTGTCGTGGAGTCGGCAATTCAGTTGAGTCACGGAT	ACACTCCAGCTGGGTGGATTTCCCAAGAT
miR-9a-3p	CTCAACTGGTGTCGTGGAGTCGGCAATTCAGTTGAGCCTCCGGT	ACACTCCAGCTGGGATAAAGCTAGGTTAC
miR-183	CTCAACTGGTGTCGTGGAGTCGGCAATTCAGTTGAGCCGTGAAT	ACACTCCAGCTGGGAATGGCACTGGTAGAAT
miR-200a	CTCAACTGGTGTCGTGGAGTCGGCAATTCAGTTGAGGACATCTT	ACACTCCAGCTGGGTAATACTGTCAGGTAAA
novel-1	CTCAACTGGTGTCGTGGAGTCGGCAATTCAGTTGAGGCGGAATC	ACACTCCAGCTGGGAGGCGAGCCTAAACGA
novel-3	CTCAACTGGTGTCGTGGAGTCGGCAATTCAGTTGAGGCAGGTAG	ACACTCCAGCTGGGTGCCGTCACAAGGACT
novel-10	CTCAACTGGTGTCGTGGAGTCGGCAATTCAGTTGAGGATACTGG	ACACTCCAGCTGGGTGCACCAAACTAATGCC
miRNA reverse primer	TGGTGTCGTGGAGTCG	
*U6* forward primer	TTGCTTCGGCGGTACATATA	
*U6* reverse primer	ATTTGCGTGTCATCCTTGC	

**Table 2:** Counts of filtered sequencing reads (in millions) for the small RNAs from different tissues.

Libraries	Ad1	Ad2	Gi1	Gi2	Ov1	Ov2	Ma1	Ma2	Total
Raw reads	1.96	5.14	13.59	5.74	6.74	3.04	5.57	8.54	50.32
Step 1	1.58	4.61	11.36	5.38	5.63	2.7	3.64	6.82	41.72
Step 2	1.57	4.61	11.35	5.37	5.63	2.7	3.64	6.81	41.68
Step 3	1.33	3.59	9.42	4.31	3.97	1.92	3.07	5.65	33.26

Step 1: Remove adapter sequences and remaining reads of 15–31 nt in length. Step 2: Remove known RNAs. Step 3: Remove unmapped reads from the reference genome.

## Supplementary Materials

The following are available online at https://www.mdpi.com/2079-7737/8/2/47/s1, Figure S1: Length distribution of filtered reads in *P. fucata*. Figure S2: The sequence composition of miRNAs in *P. fucata* displays a first position nucleotide bias for uracil (U). Figure S3: Venn diagram of the number of identified miRNAs in *P. fucata*. Figure S4: PCA of miRNA expression patterns in *P. fucata*. Figure S5. Highly expressed miRNAs in *P. fucata* mantle tissues identified by high-throughput sequencing. Table S1: Detailed information miRNAs identified in *P. fucata* by high-throughput sequencing. Table S2: Read counts and normalized RPM of miRNA expression in *P. fucata* by high-throughput sequencing. Table S3: miRNA functioning in biomineralization as predicted by RNAhybrid, miRanda, and RNA22.
