# Supplementary material for: Identification and Characterization of microRNAs and Their Predicted Functions in Biomineralization in the Pearl Oyster (Pinctada fucata)
Source: Biology (Basel). 2019 Jun 17;8(2):47. doi: 10.3390/biology8020047 (PMC6627748; doi:10.3390/biology8020047)
Supplement: Supplementary file 1 [file biology-08-00047-s001.zip › Supplementary Materials/Supplementary materials.docx]

**Identification and characterization of microRNAs and their predicted functions in biomineralization in the pearl oyster (*****Pinctada fucata*)**

**Songqian Huang^1^, Yuki Ichikawa^1^, Kazutoshi Yoshitake^1^, Shigeharu Kinoshita^1^, Yoji Igarashi^1^, Fumito Omori^2^, Kaoru Maeyama^2^, Kiyohito Nagai^3^, Shugo Watabe^4^, Shuichi Asakawa^1, *^**

1. Graduate School of Agricultural and Life Sciences, the University of Tokyo, Bunkyo-ku, Tokyo 113-8657, Japan; [huangsongqian0115@gmail.com](mailto:huangsongqian0115@gmail.com) (S.H.); [dedeneko@gmail.com](mailto:dedeneko@gmail.com) (Yu.I.); [akyoshita@g.ecc.u-tokyo.ac.jp](mailto:akyoshita@g.ecc.u-tokyo.ac.jp) (K.Y.); akino@mail.ecc.u-tokyo.ac.jp (S.K.); [aiga@mail.ecc.u-tokyo.ac.jp](mailto:aiga@mail.ecc.u-tokyo.ac.jp) (Yo.J.).
2. Mikimoto Pharmaceutical CO., LTD., Kurose 1425, Ise, Mie 516-8581, Japan; [oomori.353@mikimoto-cosme.com](mailto:oomori.353@mikimoto-cosme.com) (F.O.); [maeyama.511@mikimoto-cosme.com](mailto:maeyama.511@mikimoto-cosme.com) (K.M.).
3. Pearl Research Laboratory, K. MIKIMOTO & CO., LTD., Osaki Hazako 923, Hamajima, Shima, Mie 517-0403, Japan; [k-nagai@mikimoto.com](mailto:k-nagai@mikimoto.com)
4. School of Marine Biosciences, Kitasato University, Minami-ku, Sagamihara, Kanagawa 252-0313, Japan. [swatabe@kitasato-u.ac.jp](mailto:swatabe@kitasato-u.ac.jp)

***** Corresponding author: asakawa@mail.ecc.u-tokyo.ac.jp; Tel: +81-3-5841-5296; Fax: +81-3-5841-8166





**Figure S1.** Length distribution of filtered reads in *P. fucata*. (a) Two adductor muscle (Ad) libraries; (b) two gill tissue (Gi) libraries; (c) two ovary tissue (Ov) libraries; (d) two mantle tissue (Ma) libraries.


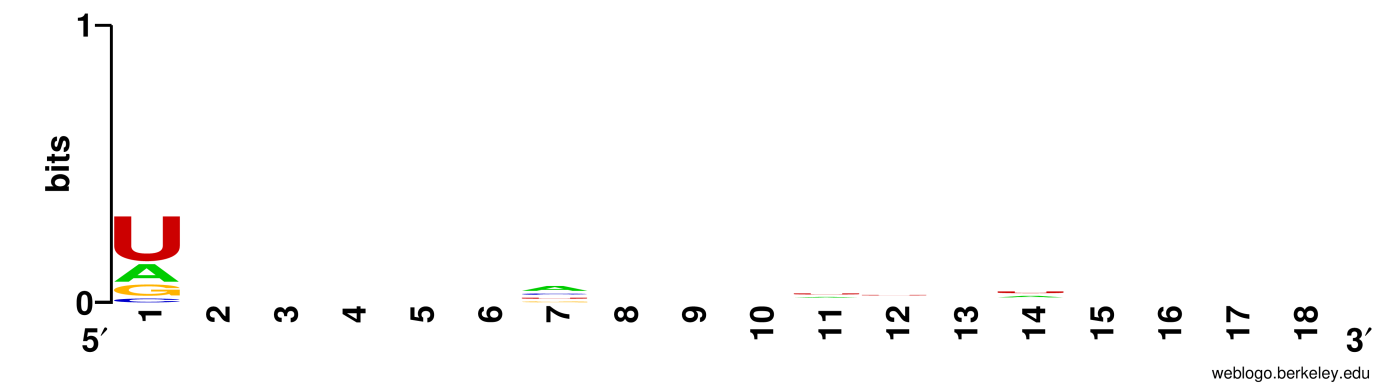


**Figure S2.** The sequence composition of miRNAs in *P. fucata* display a first position nucleotide bias for uracil (U).


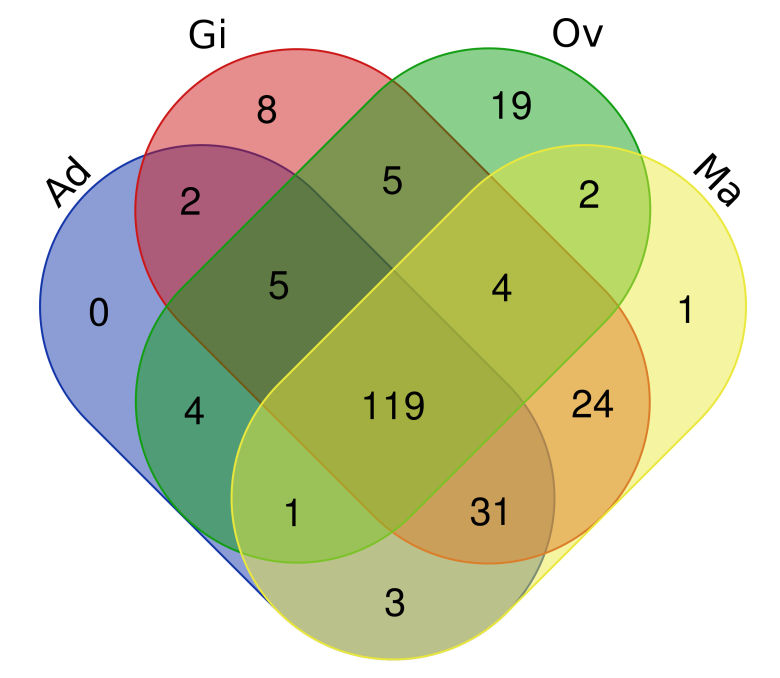


**Figure S3.** Venn diagram of the number of identified miRNAs in *P. fucata*. 165, 198, 159, and 185 miRNAs were expressed in adductor muscle, gill, ovary and mantle tissues, respectively. 119 miRNAs (52.19% of total miRNAs) were simultaneously expressed in four tissues, and 19 miRNAs (11.95% of ovary miRNA) were uniquely expressed in ovary tissues. Ad: Adductor muscle; Gi: Gill tissue; Ov: Ovary; Ma: Mantle tissue.


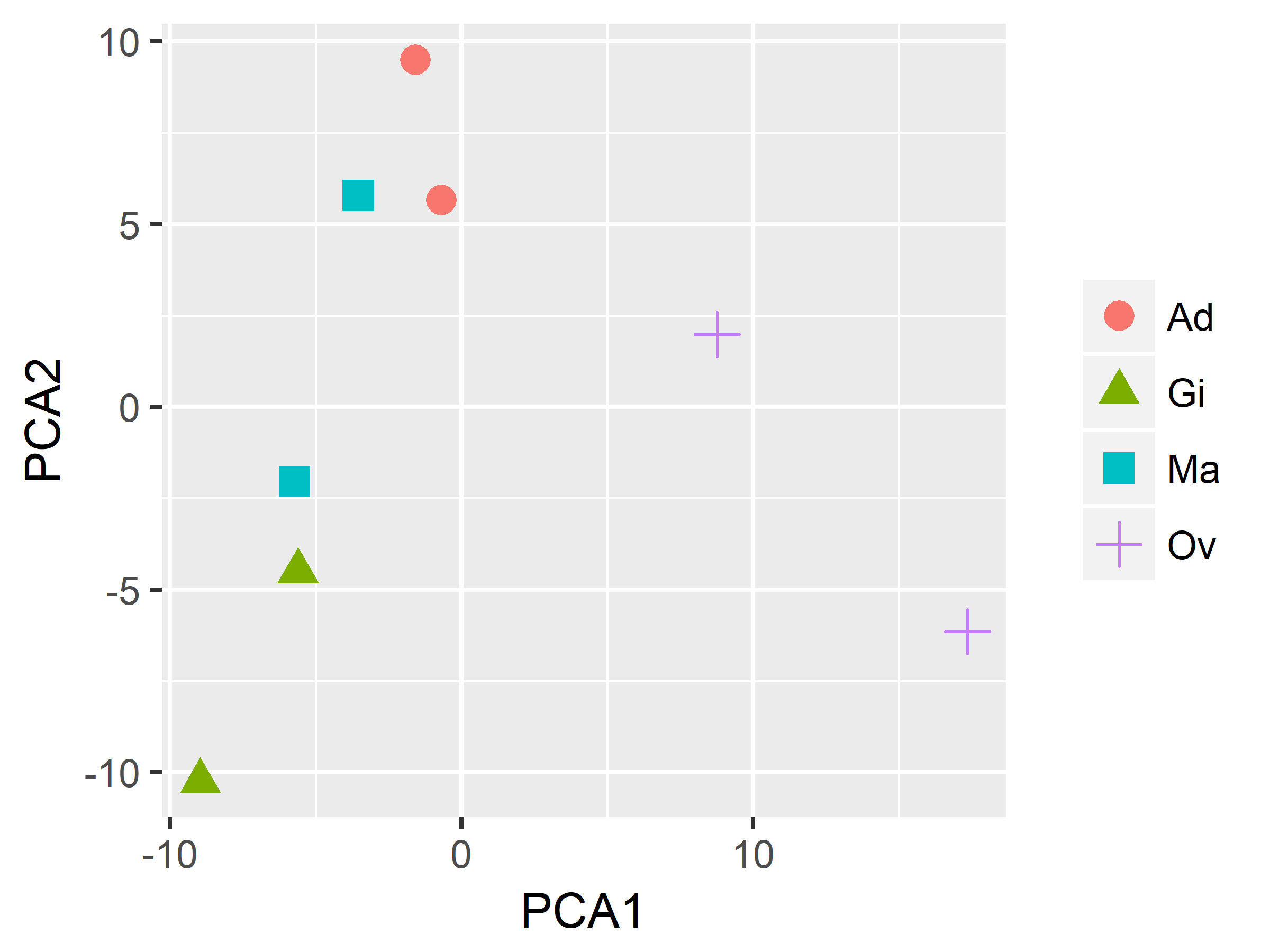


**Figure S4.** PCA of miRNA expression patterns in *P. fucata*. Ad: Adductor muscle; Gi: Gill tissue; Ov: Ovary; Ma: Mantle tissue.





**Figure S5.** Highly expressed miRNAs in *P. fucata* mantle tissues identified by high-throughput sequencing. Ad: Adductor muscle; Gi: Gill; Ov: Ovary; Ma: Mantle; RPM: Reads per million reads.
