# Supplementary material for: Identification and Characterization of microRNAs and Their Predicted Functions in Biomineralization in the Pearl Oyster (Pinctada fucata)
Source: Biology (Basel). 2019 Jun 17;8(2):47. doi: 10.3390/biology8020047 (PMC6627748; doi:10.3390/biology8020047)
Supplement: Supplementary file 1 [file biology-08-00047-s001.zip › Supplementary Materials/Table S3.docx]

**Table S3.** miRNAs functioning in biomineralization predicted by RNAhybrid, miRanda and RNA22

| Gene symbol | Accession ID | **Predicted miRNA:mRNA interaction** | | |
| --- | --- | --- | --- | --- |
|  |  | RNAhybrid | miRanda | RNA22 |
| ACCBP1 | DQ473430.1 |  | miR-8935 | novel-24 |
| ALP | AY653739.1 | miR-92b,novel-34 | miR-1799 | novel-25 |
| Alveoline-like | KR872410.1 | miR-10820,miR-154b,miR-1985,miR-3374-3p,miR-81c, miR-92a, miR-92b, miR-92c, miR-9785 | miR-12 | miR-154b,miR-2427,miR-2780d,miR-502a-3p,miR-9007,miR-92a,miR-92b,miR-92c |
| AP-1 | KP347629.1 | miR-31,miR-34a,miR-96a | miR-283,miR-43,miR-96,miR-96a,miR-96b,miR-96c,novel-8 | miR-1799,miR-4044a,miR-4044b |
| Aspein | AB094512.1 |  | miR-183,miR-200a,miR-3086-3p,miR-33,miR-4452,miR-451b,miR-8253-3p | miR-10763,miR-1671,miR-6576-3p,miR-7386i,novel-23 |
| BAMBI | KF280237.1 |  | miR-11651-3p,miR-133b,miR-2059,miR-4011a-3p,miR-7398j-3p,novel-17,novel-24,novel-40,novel-41 | miR-2059,miR-22-3p,miR-4011a-3p,miR-43e,miR-6562,miR-6562,miR-9322,novel-18 |
| BMP2 | AB176952.2 | miR-133a,miR-7386i | miR-200a,miR-216,miR-252a,miR-8871,novel-17 | miR-10265-3p,miR-12128,miR-12352,miR-184-3p,miR-2a,miR-2b,miR-2g,miR-463-3p |
| BMP3 | KT956999.1 | miR-6562,miR-7393-3p,miR-9785 | let-7a,miR-183,miR-193a,miR-4011a-3p,miR-4452,miR-5853,miR-6562,miR-7393-3p,miR-9007,novel-28 | miR-133a,miR-138-3p,miR-31,miR-317,miR-3471,miR-503,miR-503,miR-601,miR-6562,miR-7393-3p,miR-92b,miR-9322,novel-32,novel-40 |
| BMP7 | KC881250.1 | miR-12221,novel-11 | miR-1338,miR-1422p,miR-154b,miR-1728,miR-1782,miR-2059,miR-2424,miR-2427,miR-307a,miR-307b,miR-43b,miR-6545,miR-7171,novel-32,novel-39 | let-7a,miR-10723,miR-12128,miR-12128,miR-1338,miR-1776,miR-183,miR-184-3p,miR-200a,miR-2059miR-2780d,miR-286,miR-317,miR-34a,miR-34a,miR-4044a,miR-4044b,miR-6545,miR-6562,miR-71,miR-7393,miR-8935,miR-8935,miR-9007,miR-9322,miR-9322,miR-93-3p,miR-9542a-3p,novel-20,novel-31,novel-34,novel-36 |
| BMPR1b | KF280238.1 | miR-87b | miR-193a,miR-283,miR-7393 | miR-12,miR-1799,miR-183,miR-2b,miR-2c,miR-2h,miR-2j,miR-3109,miR-34a,miR-503,miR-9007,novel-32 |
| Calconectin | DQ352042.1 | miR-2059,miR-31,miR-7386i | miR-3374-3p |  |
| Calmodulin | AY341376.1 | miR-210-3p,miR-31,miR-8908b | miR-33,miR-8871 |  |
| Calreticulin | EF551334.1 |  | miR-1782 | miR-10723,miR-1782,miR-210-3p,miR-33,miR-43e,miR-4452 |
| CaM | EU921667.1 | miR-12128,miR-1671,miR-4044a,miR-4044b,miR-6562, novel-25 | miR-2285cd,miR-307a,miR-307b | let-7a,miR-12128,miR-31,miR-34a,miR-4044a,miR-4044b |
| Cathepsin-B | HQ845754.1 | miR-1-3p | miR-87a,miR-87b | miR-10723,miR-200a,miR-71,miR-7393-3p,novel-32 |
| CBP | KJ930034.2 | miR-96,miR-96a,miR-96c | miR-1671,miR-1782,miR-183,miR-200a,miR-317,miR-8935,miR-96,miR-96a,miR-96b,miR-96c | let-7a,miR-10397,miR-133b,miR-1671,miR-1782,miR-1799,miR-183,miR-184-3p,miR-210-3p,miR-34a,miR-4011a-3pmiR-4524a,miR-619-3p,miR-6562,miR-6562,miR-6770,miR-8935,miR-96,miR-96a,miR-96c,novel-15,novel-19,novel-20,novel-38 |
| Chitinase1 | KT956975.1 | miR-1799 | miR-2780d,miR-283,miR-8871,novel-16 | novel-40 |
| CLP | AY663847.1 |  |  |  |
| CMP | KU553266.1 |  |  |  |
| Dermatopontin | JQ734542.1 |  | miR-43,miR-71,novel-18 |  |
| DLX | KX889394.1 |  | miR-1493,miR-8063,miR-87a,miR-87b | miR-1422p,miR-200a,miR-2427,miR-278,miR-4011a-3p,miR-503,miR-5853,miR-5853,miR-619-3p,miR-7024-3p,novel-9 |
| EFCBP | DQ494416.1 | miR-154b |  |  |
| Fam20c | MF785096.1 |  | miR-1993,miR-2059,miR-2285cd,miR-2973-3p,miR-2a,miR-2b,miR-2c,miR-2d,miR-2e,miR-2f,miR-2g,miR-2h,miR-2i,miR-2j,miR-2k,miR-2l,miR-4011a-3p,miR-43,miR-6545,miR-8253-3p,miR-876,novel-17,novel-20 | miR-10262-3p,miR-10262-3p,miR-12128,miR-154b,miR-1728,miR-1799,miR-1993,miR-286,miR-2j,miR-31,miR-31miR-3471,miR-34a,miR-34a,miR-34a,miR-4044a,miR-4044b,miR-4185,miR-43e,miR-463-3p,miR-503,miR-5853,miR-6562,miR-6562,miR-6562,miR-6576-3p,miR-7421,miR-872,miR-87b,miR-87b,miR-9542a-3p,miR-96b,novel-14,novel-36 |
| Ferritin-like | AF547223 | novel-25 |  |  |
| GRMP | AF516712.1 |  |  |  |
| GRP | D86073.1 | miR-11268,miR-133a,miR-190-3p,miR-210-3p,miR-278, miR-9007, miR-9007, miR-9198c, miR-93-3p, miR-93-3p, miR-93-3p | miR-200a,miR-283,miR-43,miR-43b,miR-7386i,miR-8935 | miR-1985,miR-2a,miR-2b,miR-2f,miR-2g,miR-2h,miR-2j,miR-2k,miR-876,miR-8908b,novel-38 |
| Insoluble protein | D86074.1 | novel-25 | miR-11268,miR-200a,miR-7421,novel-23,novel-39 | let-7a,miR-10397,miR-1338,miR-154b,miR-154b,miR-1799,miR-1799,miR-184-3p,miR-184-3p,miR-184-3pmiR-184-3p,miR-286,miR-31,miR-31,miR-31,miR-33,miR-34a,miR-4057,miR-463-3p,miR-463-3p,miR-485-3p,miR-503,miR-6562,miR-6562,miR-6562,miR-6562,miR-71,miR-7421,miR-9681b,novel-25,novel-34,novel-38 |
| KRMP1 | DQ114788.1 | miR-10723 |  | miR-10723 |
| KRMP2 | DQ114789.1 | novel-23 | miR-7393 | miR-10262-3p |
| KRMP3 | DQ114790.1 |  | miR-7393 | miR-200a |
| Linkine | EF183520.1 | miR-1985,miR-96c,novel-3 |  |  |
| metallothionein | KC197172.1 |  | miR-503 |  |
| metallothionein2 | KC832833.1 | miR-34a,novel-19,novel-5,novel-9 | miR-1788b-3p,miR-31,miR-34a | miR-10820,miR-11268,miR-1985,miR-96,novel-34,novel-9 |
| MMP | KC881251.3 | miR-3086-3p,miR-9007,miR-93-3p,novel-14 | miR-133b,miR-1990c-3p,miR-252a,miR-2767,miR-3086-3p,miR-502a-3p,miR-93-3p,novel-24,novel-39 | miR-10723,miR-154b,miR-1799,miR-181d-3p,miR-210-3p,miR-22-3p,miR-278,miR-2l,miR-34a,miR-503,miR-71miR-872,miR-872a,miR-87a,miR-87b,miR-9007,miR-9322,miR-96a,miR-96b,novel-14,novel-24,novel-3,novel-31,novel-37 |
| mpn88 | AB295108.1 | miR-463-3p |  | miR-872 |
| mpn88-lack1 | AB295109.1 | miR-463-3p |  | miR-872 |
| mpn88-lack2 | AB295110.1 | miR-463-3p |  | miR-872 |
| mpn88-lack3 | AB295111.1 | miR-463-3p |  | miR-872 |
| mpn88-lack4 | AB295112.1 | miR-463-3p |  | miR-872 |
| mpn88-lack5 | AB295113.1 | miR-463-3p |  | miR-872 |
| mpn88-lack6 | AB295114.1 | miR-463-3p |  | miR-872 |
| mpn88-lack7 | AB295115.1 | miR-463-3p |  | miR-872 |
| MSI25 | AB210136.1 | miR-3471 | miR-1422p,miR-9692-3p | miR-1671,miR-3471,miR-485-3p |
| MSI31 | AB661680.1 |  |  |  |
| MSI60 | AB683053.1 |  |  |  |
| MSI60RP | AB689024.1 | miR-1671 |  | miR-10820,miR-1799,miR-181d-3p,miR-196a-3p,miR-29a-3p,miR-29b-3p,miR-2a,miR-2b,miR-2d,miR-2g,miR-2hmiR-2j,miR-2k,miR-2l,miR-307a,miR-307b,miR-43b,miR-463-3p,miR-6562,miR-71,miR-8215b-3p,miR-9322,novel-6 |
| MSI80 | AB683051.1 | miR-1671,miR-3471 |  | miR-1671,miR-3471 |
| N14#1 | AB023067.1 | miR-96a | miR-10311,novel-42 | miR-503,miR-6562,miR-96a,miR-96b,miR-96c |
| N14#2 | AB023249.1 | miR-12221,novel-12 | miR-10311 | miR-1671,miR-1671,miR-184-3p,miR-4057,miR-5853,miR-71,miR-7386i |
| N14#3 | AB023250.1 | miR-96a | miR-10311,novel-42 | miR-503,miR-6562,miR-96a,miR-96b,miR-96c |
| N14#4 | AB023251.1 | miR-31,miR-96a,miR-96a | miR-3481,novel-28 | miR-133b,miR-1671,miR-96a,miR-96b,miR-96c |
| N14#5 | AB023252.1 | miR-12221 | miR-10311 | miR-1671,miR-1671,miR-184-3p,miR-5853,miR-71,miR-7386i |
| N14#7 | AB023254.1 | miR-12221 | miR-10311 | miR-1671,miR-1671,miR-184-3p,miR-5853,miR-71,miR-7386i |
| N16-6 | AB808591.1 |  |  | miR-4452 |
| N16-7 | AB781153.1 |  | miR-10311 |  |
| N19 | AB332326.1 | miR-87a,miR-87b | miR-1985,miR-252a,miR-4132,miR-43e,miR-7398j-3p,novel-37 | miR-1493,miR-2l,miR-3109,miR-503 |
| N19-2 | AB781154.1 | miR-252a,novel-34 | miR-1985,miR-252a,miR-43e | miR-1671,miR-34a,miR-6562 |
| N45 | FJ913472.1 | miR-12352,miR-7421 |  |  |
| N66 | AB032613.1 | miR-1671,miR-1776,miR-34a,miR-34a,miR-503, miR-6767, miR-6770, miR-6770a, miR-8451-3p, miR-8871, novel-19 | miR-1422p,novel-19 | miR-8451-3p,miR-8871 |
| Nacrein | D83523.1 | miR-133b | miR-1422p,miR-1993,miR-283,miR-4011a-3p,miR-43b,miR-7393-3p,novel-28 | let-7a,miR-10262-3p,miR-133b,miR-2780d,miR-4011a-3p,miR-463-3p,miR-463-3p,miR-7393-3p,miR-87a,novel-25,novel-33 |
| Nacrein-B2 | HQ896199.1 |  |  |  |
| OCT1 | KM588196.1 | miR-1991 | miR-137-3p,miR-2780d,miR-33,miR-43,miR-9649,novel-28 | miR-1788b-3p,miR-503,miR-549a,miR-6770,miR-6770a,miR-7398j-3p,novel-18 |
| OCT4 | KM519606.1 | miR-11651-3p,miR-1422p,miR-4132,miR-9198c,miR-93-3p, miR-96a, novel-25, novel-34 | miR-1422p,miR-8063,miR-9198c,miR-96,miR-96a,miR-96b,miR-96c,novel-3,novel-17 | miR-10397,miR-190,miR-196a-3p,miR-1993,miR-2e,miR-33,miR-4078-3p,miR-463-3p,miR-485-3p,miR-503,miR-6576-3pmiR-6576-3p,miR-6767,miR-8935,miR-9198c,miR-92a,miR-96c,miR-9785,novel-18,novel-19,novel-19,novel-25,novel-38 |
| pearlin | AB020779.1 | miR-12221 | miR-10311 | miR-1671,miR-1671,miR-184-3p,miR-5853,miR-71,miR-7386i |
| PfCHS1 | AB290881.1 | miR-124,miR-34a,miR-96a,novel-7 | miR-124,miR-1728,miR-3771-3p,miR-71,miR-7386i,miR-8451-3p,miR-8935,novel-7,novel-20 | miR-3471,miR-96a,miR-96b,miR-96c,let-7a,miR-10723,miR-1671,miR-184-3p,miR-190,miR-210-3p,miR-2780dmiR-4011a-3p,miR-4057,novel-25 |
| PFMG1 | DQ104255.1 | miR-4057 | miR-307a,miR-307b,miR-4057,miR-8908b,novel-5 |  |
| PFMG10 | DQ116437.1 | miR-10262-3p,miR-6545,novel-2,novel-34 | miR-7393-3p | miR-10262-3p,miR-1338,miR-133a,miR-154b,miR-1776,miR-22-3p,miR-34a,miR-4181,miR-43e,miR-872,miR-872amiR-9322,novel-2,novel-2,novel-24,novel-25,novel-34,novel-38 |
| PFMG11 | DQ116438.1 | miR-286 |  | miR-5853 |
| PFMG12 | DQ116439.1 |  | miR-1993,miR-2l,miR-872 | miR-11268,miR-34a,miR-9322,novel-14 |
| PFMG2 | DQ104256.1 | miR-11268,miR-463-3p,novel-24 | miR-12,miR-133a,miR-1985,miR-2059,miR-2424,miR-31,miR-463-3p,miR-7398j-3p,novel-13,novel-20 | miR-124,miR-6707,miR-6707a,miR-9681b,miR-96a,novel-25 |
| PFMG3 | DQ104257.1 | miR-2e,miR-2e,miR-502a-3p,miR-5853,miR-7386i | miR-502a-3p | miR-6707,miR-6707a |
| PFMG4 | DQ104258.1 |  |  |  |
| PFMG5 | DQ104259.1 |  |  | miR-1493 |
| PFMG6 | DQ104260.1 |  | miR-1985,miR-71,novel-5 | let-7a,miR-190-3p |
| PFMG7 | DQ104261.1 | miR-4057 | miR-4057,miR-8908b,novel-5 | let-7a,miR-10723,miR-1671,miR-184-3p,miR-210-3p,miR-4011a-3p,miR-4057,miR-463-3p |
| PFMG8 | DQ104262.1 |  |  |  |
| PFMG9 | DQ116436.1 |  |  |  |
| PfN23 | N995665.1 | miR-1422p,miR-1799,miR-210-3p | miR-1422p,miR-2767,miR-33,miR-8871,novel-22 | miR-33,miR-6576-3p,miR-8871,novel-36 |
| PfN44 | KC238310.1 | miR-11268,miR-183,novel-28 | miR-2427,miR-4078-3p,miR-496a,miR-8871,novel-20 | miR-1493,miR-8871 |
| PfTy | AB353113.1 | miR-10262-3p,miR-183,miR-22-3p,miR-34a,miR-601, miR-96a, novel-31 | miR-2774b,miR-33,miR-3471,miR-4452,miR-8935,novel-8,novel-30,novel-36 | miR-10397,miR-10397,miR-12128,miR-154b,miR-183,miR-190-3p,miR-210-3p,miR-22-3p,miR-33,miR-34a,miR-4044amiR-4044b,miR-4057,miR-5853,miR-6757-3p,miR-9007,miR-9198c,miR-92a,miR-92b,miR-92b,miR-93-3p,novel-36 |
| Pfty1 | AB254132.1 | miR-10262-3p,miR-190-3p,miR-463-3p,miR-7386i,miR-96a, miR-96c | miR-8935 | miR-10723,miR-2427,miR-3374-3p,miR-7393-3p,novel-2,novel-36 |
| Pfty2 | AB254133.1 | miR-1671,miR-8908b,novel-15,miR-10311,miR-124, miR-196a-3p, miR-2059, miR-2780d, miR-2a, miR-2b, miR-2c, miR-2k, miR-307b, miR-3594, miR-87a, novel-28 | miR-3481,miR-4011a-3p,miR-124 | miR-8908b,miR-2973-3p,novel-19,novel-38 |
| PfY2 | KY436033.1 |  |  |  |
| Pif177 | AB236929.1 | miR-6549-3p | miR-3086-3p,miR-3771-3p,miR-43,miR-7393 | miR-11268,miR-124,miR-1671,miR-1799,miR-2774b,miR-7386i |
| Pif177-like | HE610401 |  | miR-216,miR-3481,miR-43b,miR-99a-3p,miR-99b-3p,miR-9a-3p | let-7a,miR-124,miR-1338,miR-137-3p,miR-2767,miR-2a,miR-2g,miR-33,miR-503,miR-71,miR-7421miR-99a-3p |
| PMMG1 | FJ386386.1 | miR-1664-3p | miR-2767,miR-876 | miR-1664-3p,miR-2767,miR-4057 |
| Prisilkin-39 | EU921665.1 | miR-7386i | miR-7386i |  |
| Prismalin-14 | AB159512.1 | miR-10723,miR-12221,miR-34a,miR-463-3p,miR-5853, miR-71, miR-876, miR-87b | miR-191b | miR-10723,miR-278,miR-876 |
| Prismin_1 | AB368930.2 | miR-8451-3p | miR-1-3p,miR-43 | miR-1993,miR-9335 |
| Prismin_2 | AB433980.2 | miR-8451-3p,miR-96c,novel-31 | miR-43 | miR-200a,miR-96a |
| SGMP1 | AB689023.1 | miR-1990c-3p,miR-34a,miR-71,novel-11 | miR-1-3p,miR-1422p,miR-216,miR-2l,miR-71,miR-7393-3p,novel-8,novel-11 | miR-252a,miR-34a,miR-4185,miR-6549-3p,miR-6562,miR-6576-3p,miR-7171 |
| shematrin-1 | AB244419.1 | miR-1493,miR-502a-3p |  |  |
| shematrin-2 | AB244420.1 |  | miR-283,miR-43,miR-43b,miR-8935 | miR-876,novel-38 |
| shematrin-2beta | KJ664800.1 | novel-25 | miR-283,miR-43,miR-8935 | miR-10723,miR-6770,miR-6770a,miR-876,novel-1,novel-25,novel-38 |
| shematrin-3 | AB244421.1 | miR-11268,miR-11651-3p,miR-12128,miR-12352,miR-183, miR-252a, miR-2774b, miR-4044b, miR-4132, miR-6770a, miR-7386i | miR-4132 | miR-12352,miR-3594,novel-24 |
| shematrin-4 | AB244422.1 | miR-22-3p,miR-2780d |  |  |
| shematrin-5 | AB244423.1 | miR-1799 | miR-283,novel-8 | let-7a,miR-11268,miR-1799,miR-183,miR-31,miR-6707a,miR-8451-3p,miR-981,novel-14,novel-14,novel-14novel-38 |
| shematrin-6 | AB244424.1 | miR-33 | miR-10723,miR-33,miR-7386i,miR-9322 | let-7a,miR-33,miR-3374-3p,miR-7421,miR-872,miR-872a,novel-34 |
| shematrin-7 | AB244425.1 | miR-10723,miR-183,miR-196a-3p,miR-5853,miR-7386i, miR-7393-3p | miR-196a-3p,miR-5853,novel-42 | miR-183,miR-286 |
| shematrin-8 | EF160119.1 | let-7a | miR-283,novel-34 |  |
| SPARC | AB600273.1 | miR-12221 | miR-2061,miR-43,miR-8871,novel-10,novel-12,novel-29,novel-37 | miR-317,miR-33,miR-3481,miR-34a,miR-43,miR-6545,miR-6757-3p,miR-7386i,miR-8253-3p,miR-87b,miR-9007miR-9649,miR-96b,miR-970,novel-19,novel-24 |
| TFG-β | EU137731.1 | miR-12221,miR-1422p,miR-2a,miR-2b,miR-2c, miR-2e, miR-2f, miR-2g, miR-2h, miR-2j, novel-31 | miR-1422p,miR-283 | novel-34 |
| TIMP | KC881249.1 |  | miR-4011a-3p | let-7a,miR-181d-3p,miR-96 |
| Tyr-1 | KC870906.1 | miR-4524a | miR-190,miR-3156-3p | let-7a,miR-10397,miR-10723,miR-133a,miR-286,miR-3109,miR-6637-3p,miR-7035,miR-876,novel-33,novel-5 |
| Tyrosinase | DQ112679.1 | miR-12221,miR-2061,miR-463-3p,miR-7386i | miR-3086-3p | miR-286 |
